# Untargeted Metabolomic Analysis of Cell-Free Supernatants (CFSs) from Different Clinical Isolates of *Saccharomyces cerevisiae* and Their Effects on *Candida albicans* Virulence

**DOI:** 10.3390/jof12020081

**Published:** 2026-01-23

**Authors:** Luca Spaggiari, Gabriele Tedeschi, Giulia Benatti, Michael De Benedittis, Maria Teresa Franzè, Diego Pinetti, Eva Pericolini, Andrea Ardizzoni

**Affiliations:** 1 Department of Surgical, Medical, Dental and Morphological Sciences with Interest in Transplant, Oncological and Regenerative Medicine, University of Modena and Reggio Emilia, 41124 Modena, Italy; luca.spaggiari@unimore.it (L.S.); teresa.franze@unimore.it (M.T.F.); eva.pericolini@unimore.it (E.P.); 2 Department of Life Sciences, University of Modena and Reggio Emilia, 41125 Modena, Italy; gabry.teddy@gmail.com (G.T.); giuliabenatti2003@gmail.com (G.B.); 234545@studenti.unimore.it (M.D.B.); 3 Centro Interdipartimentale Grandi Strumenti (C.I.G.S.), University of Modena and Reggio Emilia, 41125 Modena, Italy; diego.pinetti@unimore.it

**Keywords:** *Saccharomyces cerevisiae*, cell-free supernatants (CFS), *Candida albicans*, postbiotics, virulence traits, biofilm, untargeted metabolomics

## Abstract

*Saccharomyces cerevisiae* probiotic properties are effective for the treatment of infections by the opportunistic pathogen *Candida albicans*. Here, we assessed the anti-*Candida* effect of cell-free supernatants (CFSs) from three different fecal isolates and one ATCC strain of *S. cerevisiae*. We evaluated *C. albicans* growth inhibition through CFUs, and the impairment of virulence factors (adhesion, biofilm formation, and metabolic activity) by crystal violet and XTT assays. An untargeted metabolomic analysis of the CFSs was also performed. The CFSs moderately reduced *C. albicans* growth, but they could impair *C. albicans* virulence by reducing its capacity to adhere and to form a biofilm, and by decreasing the metabolic activity of biofilm-embedded fungal cells. The untargeted metabolomic analysis indicated an overexpression of N-acetyl-DL-tryptophan and other molecules derived from its metabolism (kynurenic acid and indole-3-acrylic acid), the dipeptides glycyl-L-leucine, prolyl-leucine, and γ-L-glutamyl-L-leucine, and the unconventional nucleotide inosine in the CFSs from fecal isolates, as compared to the reference strain. Further studies are warranted to better characterize the metabolome of these CFSs. Should the effects described here also be confirmed in vivo, the possible future employment of *S. cerevisiae* CFSs as a postbiotic aid to the current antifungal therapy may be considered.

## 1. Introduction

Yeasts have been used to raise bread and to produce beer and wine at least since 10,000 BC [1]. *Saccharomyces cerevisiae* (*S. cerevisiae*), one of the most commonly used, has been gradually “domesticated” by its long-lasting association with human activities, especially the fermentation processes [2,3,4,5,6]. Currently, *S. cerevisiae* is widely employed as a “cell factory” in food and pharmaceutical industries, because its metabolism can be engineered to produce a wide range of different chemicals from low-value-added, but large-volume type products (such as ethanol and biofuels) to high-value-added, but low-volume type products (pharmaceuticals) [7,8]. The presence of *S. cerevisiae* in human-related environments and its wide employment in the food industry allowed it to adapt to several body sites where it normally dwells as a commensal, such as skin [9], mucosal membranes [10,11], breast milk [12], and respiratory [13,14] and intestinal tracts [11,15,16]. These sites also offer shelter to other species of fungi, as well as to bacteria and protozoa, thus becoming colonized by a resident microbiota [17]. The rupture of such a finely balanced microbial environment may cause disease onset. Several conditions, such as Inflammatory Bowel Disease (IBD), seem to be caused by an altered mycobiota composition [15,16,18,19]. To date, scant results are available on the role of commensal fungi in these diseases. Species like *Candida albicans* (*C. albicans*) are known to live in constant interplay with the human immune system, behaving like classic opportunistic pathogens. Differently, the role of other fungi traditionally considered as commensals (such as *S. cerevisiae*) is less clear. Some studies suggest the involvement of *S. cerevisiae* in gut inflammation, because it has been found to be enriched both in stool samples of IBD patients [15,20,21] and in the gut mucosa of Crohn’s Disease (CD) patients [11]. Other studies, though, indicate a positive role for *S. cerevisiae* in the gut colonization of CD pediatric patients [16]. In addition, *S. cerevisiae*, together with the cognate species *Saccharomyces boulardii*, has been shown to exert antimicrobial activity also against pathogenic bacteria, such as some *Salmonella* serovars [22]. The idea of a beneficial role of *S. cerevisiae* on human health is also reinforced by its pharmaceutical applications as a probiotic [17]. In addition, *S. cerevisiae* metabolic products, obtained through the production of fungal cell-free supernatants (CFSs), have been demonstrated to stimulate *Bacillus subtilis* spore germination (making the bacteria more vulnerable), with important applications in the food industry, where *B. subtilis* often occurs as a contaminant [23]. Furthermore, they can inhibit the biofilm formation of *Staphylococcus aureus* [24] and *Listeria monocytogenes* [25], important contaminants greatly feared in the food industry.

The Food and Agriculture Organization of the United Nations (FAO) and the World Health Organization (WHO) revised the definition of probiotics in 2001, as follows: “probiotics are live microorganisms which, when administered in adequate amounts, confer a health benefit on the host” [26,27]. Probiotics, by definition, are alive, and they are required to have an efficacious number of viable cells when they are administered to humans. However, most probiotic preparations, especially at the end of their shelf-life, can include potentially large numbers of dead and injured microorganisms, whose potential influence on probiotic functionality has received little attention to date [28,29]. Indeed, it has been demonstrated that bacterial lysates from respiratory pathogens are effective in preventing pediatric respiratory diseases, possibly through mechanisms of trained immunity [30]; in a recent work by our group, we demonstrated that a *Cutibacterium acnes* bacterial lysate was able to improve vaginal epithelial cell response to pathogens responsible for vaginal infections [31]. Therefore, the term “postbiotic” has been introduced to indicate all those “preparations of inanimate microorganisms and/or their components (and product of their metabolism, including CFSs) that confer a health benefit on the host” [32].

*C. albicans* is a dimorphic fungus and a member of the human mycobiota [33]. In its yeast form, it behaves as a harmless commensal, and it dwells in the mucosae of the oropharynx, genital, and gastrointestinal tracts within the human body [34]. Upon immune system impairment, *C. albicans* can undergo dimorphic transition and shift to its hyphal form, becoming thus an opportunistic pathogen responsible for oral–pharyngeal candidiasis, esophageal candidiasis, genital tract infections (such as vulvovaginal candidiasis, VVC), and severe nosocomial bloodstream infections [34,35]. The colonization of the mouth, vagina and gut by *Candida* occurs very early in life, typically during infancy. Within the gut, *C. albicans* is forced into its yeast form by a competent immune system. In this form, the fungus has a positive role in shaping the composition of a healthy microbiota and in inhibiting inflammatory responses. In addition, the presence of *C. albicans* in the gut has been associated with an increase in splenic IgG-producing B cells and systemic antifungal IgG, which confer protection from candidemia [36,37,38,39,40]. *C. albicans* is a leading cause of nosocomial infections, with mortality rates often exceeding 40% despite treatment [41,42]. The handling of systemic infections by *Candida* and other fungal pathogens is complicated by the availability of only three classes of antifungal drugs, namely the azoles that target ergosterol biosynthesis, the echinocandins that inhibit fungal cell wall biosynthesis, and the polyenes that bind to ergosterol in the fungal cell membrane, leading to cell lysis [43,44]. In addition to our limited arsenal of antifungals, the development of multidrug-resistant strains and the emergence of intrinsically resistant pathogens are increasing.

Increasing evidence indicates that specific strains of *S. cerevisiae* have probiotic properties, and they have been used successfully for the treatment of bacterial and fungal infections, especially by *C. albicans* [45]. The latter and *S. cerevisiae* coexist pacifically in the gut, but in different contexts, such as in oropharyngeal candidiasis and in VVC, the use of *S. cerevisiae* as a probiotic has been demonstrated to be protective against *C. albicans* infections [46,47,48]. However, these data make use of alive yeasts as probiotics, or of dead whole yeast cells. In the present work, we prepared CFSs (containing metabolites and other molecules produced and secreted by alive yeasts) from three different fecal isolates and one ATCC reference strain of *S. cerevisiae*. We investigated the effects of CFSs on *C. albicans* growth and its capacity to adhere to abiotic substrates and to form a biofilm; in addition, we assessed whether the CFSs affect the metabolic activity of biofilm-embedded *Candida* cells. We then analyzed the overexpression of metabolites by the three *S. cerevisiae* strains isolated from human stools in comparison to the reference *S. cerevisiae* ATCC strain. By comparing our results with data from the literature, we tried to understand if the overexpression of certain metabolites could provide a potentiated activity of such CFSs against *C. albicans*.

## 2. Materials and Methods

### 2.1. Fungal Strains

**Figure 1:**
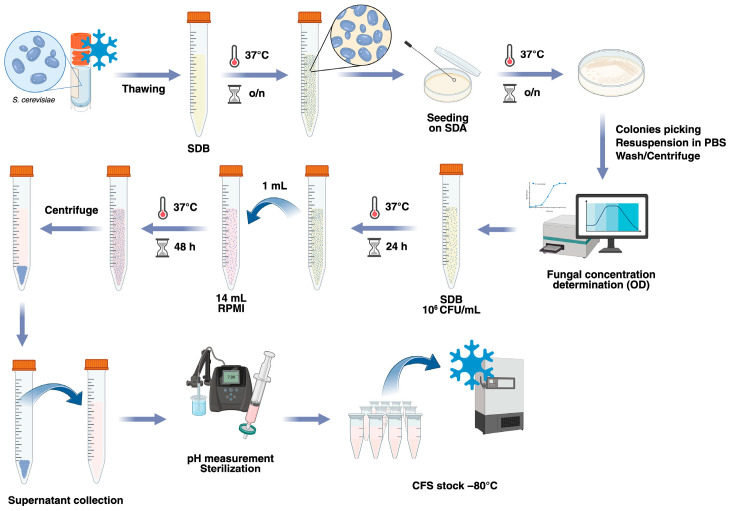
Schematization of the protocol used for the preparation of *S. cerevisiae* CFSs. *S. cerevisiae* strains were thawed, grown in SDB for 24 h, and seeded onto SDA to obtain single colonies. Isolated colonies were washed and resuspended in SDB at a concentration of 10^6^ CFU/mL and grown for 24 h. Then, 1 mL of each culture was transferred to RPMI-1640 media, where the yeasts were allowed to grow for a further 48 h. Finally, the fungal suspensions were centrifuged, and the cell-free supernatants (CFSs) were collected. After measurement of the pH and sterilization through 0.22 µm filters, the CFSs were aliquoted and stored frozen at −80 °C. Created in BioRender. Ardizzoni, A. (2026) https://BioRender.com/remublw.

Four strains of *S. cerevisiae* were selected to produce cell-free supernatants (CFSs). Strains 66, 83, and 84 were obtained by fecal isolates of 3 different healthy subjects, and they had been already genotypically and phenotypically characterized [49]. Strain 87, a reference strain (*S. cerevisiae* ISM 68/119-ATCC 9763), was purchased from LTA S.r.l. (Milano, Italy). The effects of *S. cerevisiae* CFSs were assessed on the *Candida albicans* strain SC5314 (ATCC MYA-2876). All the strains were stocked at −80 °C in cryovials containing plastic beads (Pro-Lab Diagnostics, Bromborough, UK). The fungi were thawed by collecting a single bead with a sterile inoculating loop, transferring it into Sabouraud Dextrose Broth (SDB, Condalab, Madrid, Spain), and incubating it overnight at 37 °C. Then, 10 µL of each suspension was seeded onto fresh Sabouraud Dextrose Agar (SDA, Oxoid, Fisher Scientific, Milan, Italy) plates and incubated for 24 h at 37 °C to obtain single colonies and to check the viability and purity of each strain (Figure 1).

### 2.2. Preparation of S. cerevisiae Cell-Free Supernatants (CFSs)

*S. cerevisiae* cell-free supernatants (CFSs) were prepared according to a previously described protocol [50], with minor modifications. Briefly, the *S. cerevisiae* strains were subcultured through passages in fresh SDA plates and incubated overnight at 37 °C, to have the fungi in their exponential growth phase. For each strain, a suspension of 10^6^ CFU/mL in SDB was prepared and incubated for 24 h at 37 °C. Then, 1 mL of each fungal suspension was added to tubes containing 14 mL of RPMI-1640 medium (Sigma-Aldrich, Saint-Louis, MO, USA), which were then incubated at 37 °C for 48 h. The medium RPMI-1640 was chosen according to the protocol set up by De Marco and coworkers [50]. At the end of the incubation, the concentration of each strain was measured spectrophotometrically with a Sunrise Microplate Reader (Sunrise, Tecan, Salzburg, Austria) to ascertain that the fungi continued to grow. The suspensions were then centrifuged at 3000 rpm for 10 min, and the supernatants obtained by centrifugation (i.e., the cell-free supernatants) were collected. After measuring the pH (with values ranging from 7.0 to 7.4), every CFS was sterilized by filtration through 0.22 µm filters, divided into 1 mL aliquots in cryovials, and stored at −80 °C. The protocol described is summarized in Figure 1.

### 2.3. Effects of S. cerevisiae CFSs on C. albicans Growth

The day before each experiment, *C. albicans* was subcultured onto fresh SDA plates and incubated overnight at 37 °C, allowing the fungus to reach the exponential growth phase. On the day of the experiment, a loopful of *Candida* was collected from the plate and washed in 1× PBS. Then, after spectrophotometric measurement, its concentration was assessed using a stored curve set up in our laboratory, which associates optical density (OD) values with a specific fungal concentration. The *Candida* was then diluted to a working strength concentration of 10^6^ CFU/mL. To assess *C. albicans* growth efficiency, 500 µL of fungal suspension (10^6^ CFU/mL) was added to tubes containing 500 µL of the different CFSs or RPMI (used as a negative control) and incubated at 37 °C for 24 h under agitation. The fungal suspensions were then serially diluted, and 20 µL from each dilution was seeded as spots onto SDA plates. The plates were incubated at 37 °C for 24 h to allow for fungal growth, then the CFUs were counted. The CFU values obtained from the *Candida* grown with the CFSs were normalized as percentages, compared to the CFU values of the control, i.e., *Candida* grown in RPMI-1640, which were considered 100%. CFU values similar to or higher than the CFU value of the control were assigned the 100% value as well. The data were then drawn into heatmaps that reported the mean percentage of CFU values from 5 independent experiments.

### 2.4. Effects of S. cerevisiae CFSs on C. albicans Adhesion, Biofilm Formation, and Biofilm Metabolic Activity

The day before each experiment, *Candida* was subcultured onto fresh SDA plates and incubated overnight at 37 °C, allowing the fungus to reach the exponential growth phase. A loopful of *Candida* was then collected from the plate, washed in 1× PBS, its concentration was measured by OD determination, and a suspension of 10^6^ CFU/mL was prepared.

For the assessment of adhesion, 100 µL of *Candida* suspension was added to the wells of a 96-well microtiter plate (Greiner, Milano, Italy) containing 100 µL of each CFS. As a control, 100 µL of *Candida* suspension was added to 100 µL of sterile RPMI-1640. The plate was incubated for 2 h at 37 °C with 5% CO_2_, and the adhesion was evaluated by crystal violet (CV) staining, using a protocol previously described [51]. Briefly, the supernatants were removed, each well was washed with warm PBS to remove non-adherent fungal cells, and 100 µL of the 1% crystal violet (CV, Sigma-Aldrich, St. Louis, MO, USA) was added. After 5 min, the CV was removed, and 1 wash with warm PBS was performed before adding 100 µL of 33% acetic acid (ITW Reagents, Monza, Italy) to each well. The adhesion of *C. albicans* was quantified by evaluating the optical density (OD) at the 570 nm wavelength using a spectrophotometer (Sunrise, Tecan, Männedorf, Switzerland). Each condition was tested in triplicate across 3 independent experiments.

To assess the capacity of *Candida* to form a biofilm, 100 µL of *Candida* suspension (10^6^ CFU/mL) was added to the wells of 96-well microtiter plates (Greiner) containing 100 µL of each CFS. As a control, 100 µL of *Candida* suspension was added to 100 µL of sterile RPMI-1640. The plates were incubated for 24 h at 37 °C with 5% CO_2_, and the biofilm formation was then evaluated by CV staining, using a protocol previously described [52,53,54]. Briefly, at the end of incubations, the supernatants were removed, and each well was washed 3 times with warm PBS. Then, biofilms were fixed for 15 min with 99% methanol (ITW Reagents) and stained for 5 min with 1% CV. After removal of the dye, the wells were washed with distilled water and finally treated with 33% acetic acid for 10 min to solubilize the residual staining. The biofilm of *C. albicans* was quantified by evaluating the optical density (OD) at the 540 nm wavelength using a spectrophotometer. The cut-off value for the presence of a biofilm was an optical density of at least 1.5. Each condition was tested in quadruplicate across at least 3 independent experiments.

To evaluate the metabolic activity of *C. albicans* biofilm, 100 µL of *Candida* suspension was added to the wells of 96-well microtiter plates (Greiner) containing 100 µL of each CFS. As a control, 100 µL of *Candida* suspension (10^6^ CFU/mL) was added to 100 µL of sterile RPMI-1640. The plates were incubated for 24 h at 37 °C with 5% CO_2_, and an XTT reduction assay was performed, according to previously established protocols [52,53,54], with slight modifications. Briefly, after the end of incubation, the supernatants were removed, and the wells were washed 3 times with warm PBS. Then, 100 µL of a 2,3-bis(2-methoxy-4-nitro-5-sulfophenyl)-2H-tetrazolium-5-carboxanilide (XTT) solution containing 10 mM Menadione (Sigma-Aldrich) was added to each well, and the plates were incubated for a further 2.5 h at 37 °C with 5% CO_2_. Finally, 80 µL was collected from each well, transferred to another 96-well plate, and the absorbance was measured immediately by a spectrophotometer at a wavelength of 492 nm. Each condition was tested in quadruplicate across at least 3 independent experiments.

### 2.5. Liquid Chromatography–Electrospray/High-Resolution Mass Spectrometry (HPLC-ESI/HRMS)

The CFSs, which had been stored at −80 °C, were thawed and centrifuged at 18,000× *g* for 10 min and transferred to Amicon-Ultra 0.5 tubes. Then, the HPLC-ESI/HRMS analysis was performed following the method described in a previous paper by our group [55], with minor modifications. Briefly, the amended parameters were the following: the separation was performed at 0.3 mL/min flow; the mobile phase composition, kept at 2% B for 1 min after injection, was linearly raised to 35% B in 30 min; methanol was kept at 98% up to minute 39.9 and lowered to 2% at minute 40, with a total runtime of 50 min; Sheath Gas 37 and Aux Gas 28 were the nitrogen flows used to assist the ionization; the capillary voltage was set at 3.5 kV (or 3.2 kV for negative ionization); 243 ms was the maximum injection time; finally, the fragmented precursors were dynamically excluded for 10 s.

### 2.6. Compounds Discoverer Data Analysis

Raw files (quadruplicate samples from each different CFS) were processed by Compound Discoverer (CD) 3.3.2.31 (Copyright 2014–2023 Thermo Fisher Scientific Inc., Waltham, MA, USA) using a slightly modified processing workflow template for Untargeted Metabolomics with Statistics Detect Unknowns with ID Using Local Databases, as previously described [55]. Briefly, the core of the workflow consisted of Spectra selection from raw files (Retention Time limited from 0.2 to 45 min), Retention Time Alignment (ChromAlign) with respect to a QC sample file, and Compound Detection and Grouping with RT tolerance of 0.3 min and 5 ppm mass deviation. Then, Gap Filling, SERRF QC Correction, and Background removal were performed along with Compound Annotation using Predicted Composition and different types of databases (mzCloud, mzVault, Metabolika, ChemSpider, MassList) [56]. The detected compounds were used for the differential analysis of sample groups (Nested Design; Generated Ratios: *S. cerevisiae* CFS 66/CFS Ref, CFS 83/CFS Ref, and CFS 84/CFS Ref). According to the confidence level of identification, each compound was assigned a full match (colour code: green), a partial match (colour code: orange), or no match (colour code: red) for each of the annotation sources listed above. Only those compounds that were assigned a name by the software and whose annotation sources returned at least 3 full matches, or 2 full matches plus 2 partial matches, were included in the tables. Moreover, to improve clarity, an identification level has been indicated, according to data from the literature [57].

### 2.7. Statistical Analysis

The statistical analyses have been carried out using GraphPad Prism 10 software (GraphPad, Boston, MA, USA). All the experiments have been repeated at least 3 times, and each experiment consisted of at least 3 technical replicates for every condition tested. The columns depicted in the graphs represent the average values ± SEM. The Shapiro–Wilk test was used to analyze data distribution within each experimental group. Statistical differences among groups were assessed through One-Way ANOVA, followed by Dunnet’s multiple comparisons test or by the Kruskal–Wallis test, then followed by an Uncorrected Dunn’s test. Values of *p* ≤ 0.05 were considered significant.

## 3. Results

### 3.1. CFS Production and Characteristics

Four strains of *S. cerevisiae* were employed for the present study: three fecal isolates (strains 66, 83, and 84) obtained from healthy subjects who followed Western and omnivore diets and had not been treated with prebiotics and/or probiotics for 1 month, nor with antibiotics for at least 3 months [49] and one ATCC strain (strain 87, referred to as CFS Ref). Four different batches of CFSs were prepared and employed for the experiments described. All batches were checked for the fungal concentration that had been reached before supernatant collection, and for the pH values of the supernatants. Before collecting the supernatants, the fungal growth ranged between 4.1 × 10^6^ CFU/mL and 7.8 × 10^6^ CFU/mL (with an average growth of 6.4 × 10^6^ CFU/mL), and the pH values were always very close to neutral, ranging between 7.0 and 7.2, with an average value of 7.1.

### 3.2. Effects of S. cerevisiae CFSs on C. albicans Growth, Adhesion, Biofilm Formation and Metabolic Activity of Biofilm-Embedded Fungi

**Figure 2:**
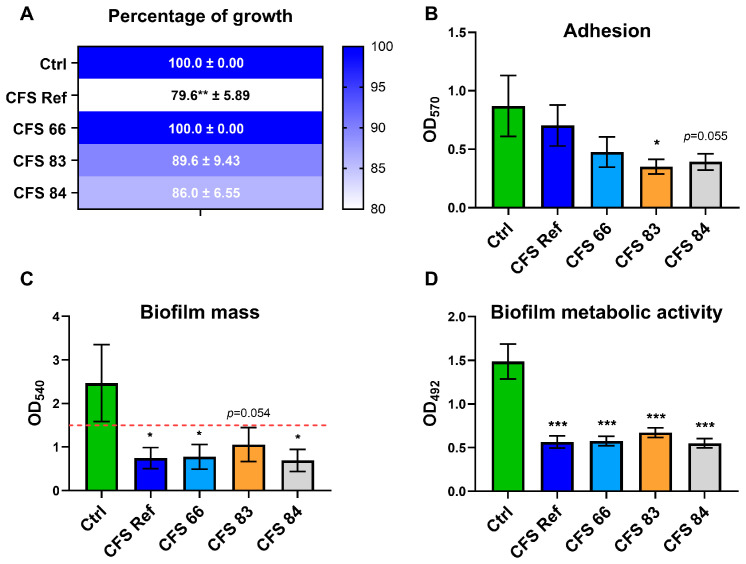
Effects of *S. cerevisiae* CFSs on *C. albicans* growth, adhesion, biofilm formation, and metabolic activity. (**A**) *C. albicans* SC5314 grown onto SDA plates, after 24 h of incubation with, or without (Ctrl), *S. cerevisiae* CFSs (CFSs 66, 83, 84, and Ref). The CFU values have been normalized and expressed as percentages relative to the CFU value of the control, which has been arbitrarily assigned the value of 100%. The values are represented with heatmaps with colours shading from blue (high percentages) to white (low percentages). ** *p* ≤ 0.01. (**B**) Adhesion evaluated after *C. albicans* SC5314 incubation for 2 h with, or without (Ctrl), the CFSs (CFSs 66, 83, 84, and Ref). * *p* ≤ 0.05. (**C**) Biofilm formation assessed after *C. albicans* SC5314 incubation for 24 h with, or without (Ctrl), the CFSs (CFSs 66, 83, 84, and Ref). * *p *≤ 0.05. (**D**) Metabolic activity of biofilm-embedded *C. albicans* SC5314 cells after 24 h of incubation with, or without (Ctrl), the CFSs (CFSs 66, 83, 84, and Ref). *** *p* ≤ 0.001.

To evaluate the effects of the CFSs on *Candida* growth, *C. albicans* SC5314 was incubated with the different CFSs for 24 h, then seeded onto SDA plates. For every condition, the CFU values counted were expressed as a percentage of the CFU value of the control (considered as 100% growth) and the results were reported on heatmaps. After 24 h, the growth of *Candida* was not affected by incubation with CFS 66, while the incubation with the other CFSs exhibited a reduction in fungal growth. Such growth reduction reached statistical significance when *C. albicans* was incubated with CFS Ref (Figure 2A).

Moreover, after 2 h of contact with the CFSs, a reduced adhesion capacity of *C. albicans* was observed after incubation with all but CFS Ref, when compared to the control. Notably, such reduction reached statistical significance only for *C. albicans* incubated with CFS 83 (Figure 2B). Upon extending the CFS contact time to 24 h, in order to allow *C. albicans* to form a biofilm, the fungal cells treated with all the CFSs exhibited an impairment in their biofilm formation capacity: crystal violet staining returned OD values lower than the cutoff value of 1.5; in contrast, the control *C. albicans* cells, grown without CFSs, were able to form a biofilm, as shown by the OD values well above the cut-off value (Figure 2C). To determine whether the impairment of biofilm formation was due to a reduction in fungal metabolic activity, an XTT assay was performed. The results show that 24 h contact with the CFSs impaired the metabolic activity of biofilm-embedded *C. albicans* cells compared to the control, and this impairment reached statistical significance across all *C. albicans* cells incubated with CFSs (Figure 2D).

### 3.3. Untargeted Metabolomic Analysis of CFSs from S. cerevisiae

**Figure 3:**
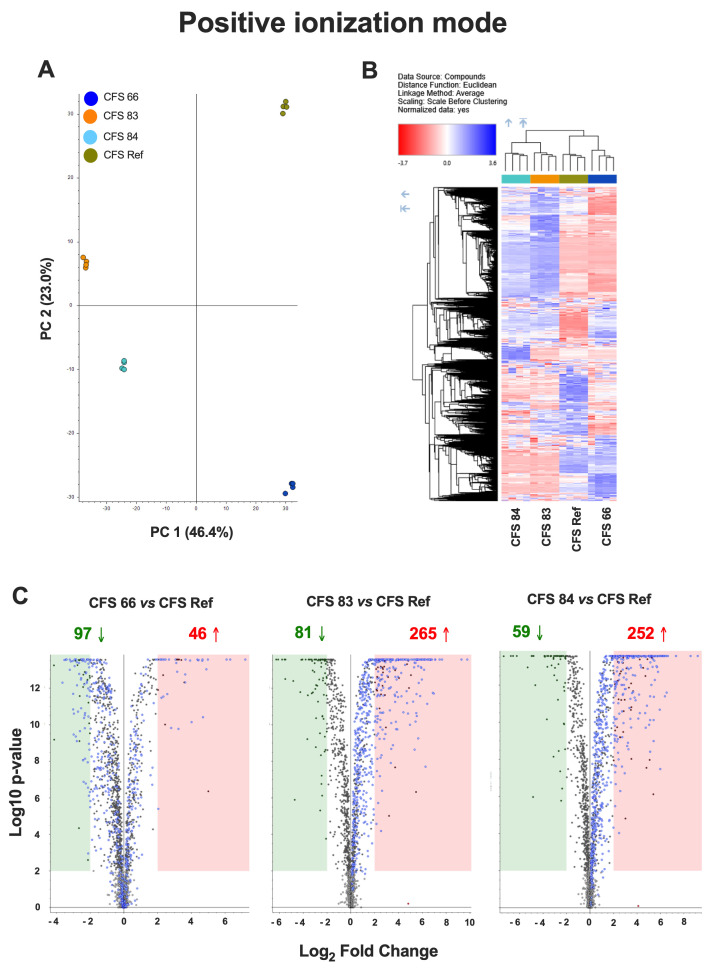
(**A**) Principal Component Analysis (PCA) of the metabolomes from CFSs 66, 83, 84, and Ref, analyzed in positive ionization mode. (**B**) Hierarchical clustering analysis in positive ionization mode, carried out to compare the metabolomes from CFSs 66, 83, 84, and Ref, according to Compound Discoverer (CD) 3.3.2.31 analysis. The blue regions include clusters of overexpressed metabolites, and the red regions include clusters of downregulated metabolites in CFSs 66, 83, 84, and Ref. (**C**) Chart maps of significant (*p* < 0.01, Log_2_ Fold Change = 2) upregulated (red) or downregulated (green) metabolites in CFSs 66, 83, and 84 as compared to CFS Ref analyzed in positive ionization mode. Data are from quadruplicate samples from each CFS.

**Figure 4:**
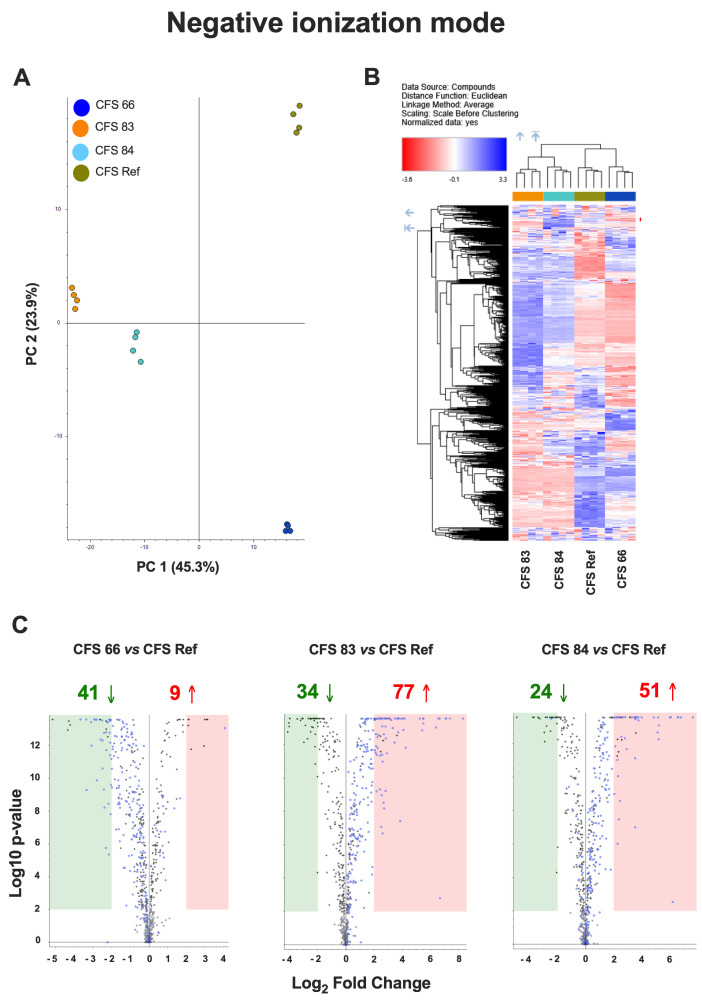
(**A**) Principal Component Analysis (PCA) of the metabolomes from CFSs 66, 83, 84, and Ref, analyzed in negative ionization mode. (**B**) Hierarchical clustering analysis in negative ionization mode, carried out to compare the metabolomes from CFSs 66, 83, 84, and Ref, according to Compound Discoverer (CD) 3.3.2.31 analysis. The blue regions include clusters of overexpressed metabolites, and the red regions include clusters of downregulated metabolites in CFSs 66, 83, 84, and Ref. (**C**) Chart maps of significant (*p* < 0.01, Log_2_ Fold Change = 2) upregulated (red) or downregulated (green) metabolites in CFSs 66, 83, and 84 as compared to CFS Ref analyzed in negative ionization mode. Data are from quadruplicate samples from each CFS.

An untargeted metabolomics approach was used to compare the metabolomes of the CFSs from the different *S. cerevisiae* strains (CFSs 66, 83, 84, and Ref), both in positive and negative ionization mode. Principal Component Analysis (PCA) showed significantly different metabolomic profiles among the four CFSs (Figure 3A and Figure 4A). A hierarchical clustering analysis, carried out to compare the four metabolomes, revealed distinct clusters of metabolites overexpressed (blue areas) or downregulated (red areas) in CFSs 66, 83, 84, and Ref (Figure 3B and Figure 4B).

Chart maps obtained by positive ionization mode analysis were drawn to show the comparison of the metabolites occurring in the CFSs from *S. cerevisiae* clinical isolates with the metabolites occurring in the reference CFS. Specifically, 97 metabolites were downregulated, and 46 metabolites were upregulated in CFS 66 as compared to CFS Ref; 81 metabolites were downregulated, and 265 metabolites were upregulated in CFS 83 as compared to CFS Ref; 59 metabolites were downregulated, and 252 metabolites were upregulated in CFS 84 as compared to CFS Ref (Figure 3C). Chart maps obtained by negative ionization mode show that 41 metabolites were downregulated, and 9 metabolites were upregulated in CFS 66 as compared to CFS Ref; 34 metabolites were downregulated, and 77 metabolites were upregulated in CFS 83 as compared to CFS Ref; 24 metabolites were downregulated, and 51 metabolites were upregulated in CFS 84 as compared to CFS Ref (Figure 4C).

A more detailed analysis of the upregulated metabolites included in the specific blue areas was carried out to compare CFSs 66, 83, and 84 to CFS Ref, both in positive and negative ionization mode. The identified overexpressed compounds in these areas returned different levels of identification according to the Annotation Sources used by the CD software 3.3.2.31 and listed in Supplementary Tables S1 and S2.

According to the results of the analysis in positive ionization mode, N-acetyl-DL-tryptophan (two full matches and two partial matches) and indole-3-acrylic acid (five full matches) were upregulated in all the CFSs with respect to CFS Ref. Imidazolelactic acid (three full matches and one partial match) was upregulated only in CFS 66, and kynurenic acid (five full matches and one partial match) was upregulated only in CFS 83. Notably, in CFS 83, leucine-containing molecules (i.e., glycyl-L-leucine, prolylleucine, g-L-glutamyl-L-leucine) were also upregulated (Table S1).

The analysis in negative ionization mode returned a small number of upregulated compounds. Interestingly, inosine (three full matches and one partial match) was upregulated in CFS 84 (Table S2).

## 4. Discussion

*S. cerevisiae* and *C. albicans* often dwell in the same ecological niches within the human body, where they compete for attachment to epithelial cells [58]; such competition, if effective on the *S. cerevisiae* side, may inhibit colonization and dissemination of *C. albicans* [46]. It has been reported, in a recently published study, that not only *S. cerevisiae* per se, but also cell-free supernatants (CFSs) obtained from a combination of probiotics of bacterial and fungal origin (including *S. boulardii*) can affect *C. albicans* dimorphic transition [59].

Therefore, in the present study, we prepared CFSs from four *S. cerevisiae* strains (three fecal isolates and one ATCC strain), and we assessed their activity against *C. albicans*. Four different batches of CFSs were prepared, which were necessary to carry out the many experiments described in this paper. For every batch, the yeasts’ CFU values as well as the final CFS pH were measured, and no batch-to-batch differences were observed, which attests to the reproducibility of the method employed. In particular, the similar CFU values of each strain at the end of the 48 h incubation period indicate that the conditions chosen to grow *S. cerevisiae* allowed the yeasts to survive and reproduce, thus indicating that the CFSs mainly contain molecules originating from the metabolic activity of living cells, rather than dead cells or parts of dead cells.

pH values around 7.0, in combination with the presence of serum, low percentages of CO_2_, and other facilitating conditions, allowed *C. albicans* to undergo the dimorphic transition and to shift to its hyphal form, which is associated with fungal pathogenesis [33]. However, the neutral pH of *S. cerevisiae* CFSs was not sufficient per se to allow *C. albicans* to express its virulence. Indeed, several virulence factors of *C. albicans* were impaired, which may be ascribed to the *S. cerevisiae*-secreted molecules occurring in the CFSs, which overcome the advantage provided by the neutral pH. We initially evaluated the impairment of *C. albicans* growth; after 24 h incubation with the CFSs, none were able to stop *C. albicans* from growing. The CFSs from two out of the three fecal isolates reduced *C. albicans* growth, but this reduction did not reach statistical significance, indicating that the fungus survived and retained its ability to replicate, albeit at a lower rate. In contrast, the CFS Ref, obtained from the ATCC strain, was able to significantly reduce *C. albicans* growth after 24 h of incubation. However, the capacity to survive and to replicate did not account, per se, for fungal pathogenicity. Indeed, *Candida* virulence traits may have been affected by the incubation of the fungus with the CFSs. *C. albicans* in its hyphal form behaves as an opportunistic pathogen by adhering to biotic and abiotic substrates, and this capacity to adhere is the first step leading to biofilm formation [60]. The results of the present study show that the adhesion capacity of *C. albicans* to an abiotic substrate was impaired after 2 h of incubation with all the CFSs. Such impairment likely persisted throughout the 24 h of incubation, because the biofilm mass production remained well below the cut-off value, and the metabolic activity of the sessile fungal cells incubated with all the CFSs was significantly reduced compared to the untreated *Candida*. Therefore, notwithstanding the moderate CFS effects on *C. albicans* growth, the impairment of its virulence factors, such as the diminished capacity to adhere, the reduced ability to form a structured biofilm, and the hindered metabolic activity of biofilm-embedded fungal cells, is clinically relevant because the impairment reduces the possibility for *Candida* to exert its pathogenic activity. It follows that the CFSs must contain one or more components responsible for the observed effects. Therefore, we carried out an untargeted metabolomic analysis comparing the expression of metabolites in the CFSs from the fecal isolates versus the CFS from the reference ATCC *S. cerevisiae* strain (CFS Ref). The principal component analysis, together with the hierarchical clustering analysis of overexpressed and downregulated compounds, indicated that the metabolomic profiles of CFS 83 and CFS 84 were more similar, whereas the metabolomic profile of CFS 66 was more similar to the metabolomic profile of CFS Ref. The analysis of the single compounds within the hierarchical clustering showed an upregulation of N-acetyl-DL-tryptophan and other molecules derived from its metabolism (kynurenic acid and indole-3-acrylic acid) in the CFSs from fecal isolates. Kynurenic acid increases in osmotic stress conditions [61]; it has a variety of cytoprotective, neuroprotective, and neuronal signalling properties, and it is the terminal element of an irreversible reaction of most tryptophan degradation pathways. Interestingly, kynurenic acid has been shown to be potentially useful as a nutraceutical [62]. The literature reports several properties for indoles, such as antioxidant, anti-inflammatory, and neuroprotective activity. Indole-3-acrylic acid [63,64] has also been reported to promote intestinal epithelial-barrier function and to mitigate inflammatory responses. Recently, indole and its derivatives have also been reported to inhibit the filamentation and biofilm formation of *C. albicans*, which may partly explain the inhibition of *C. albicans* virulence traits by *S. cerevisiae* CFSs [63,65]. Inosine, a metabolite upregulated in the CFS from strain 84, is a non-canonical nucleotide that exerts antioxidant, anti-inflammatory, and neuroprotective effects [66], and it has a positive impact both on the host and on its resident microbiota. Inosine has antioxidant, anti-inflammatory, pro-axogenic, and neuroprotective functions, and for these reasons, it is also employed as a therapeutic supplement for nerve injury, inflammation, and oxidative stress [66,67]. In CFS 83, an upregulated production of glycyl-L-leucine, prolyl-leucine, and γ-L-glutamyl-L-leucine was observed. These dipeptides primarily serve as precursors of their constituent amino acids. Leucine, the common component of these dipeptides, is an essential branched-chain amino acid that is vital for human health as it cannot be synthesized by the body and must be obtained from one’s diet. Indeed, several γ-glutamyl compounds, including γ-L-glutamyl-L-leucine, are widely distributed in many animal and vegetal tissues [68]. Leucine has many biological properties, and it is involved in protein metabolism, energy regulation, and cellular signalling. Indeed, leucine has been reported to have a positive effect on lipid metabolism and insulin sensitivity and, therefore, was proposed as a potential agent for the prevention and treatment of metabolic diseases, including obesity and type 2 diabetes [69]. It follows that the overproduction of this metabolite (and/or its precursors) by specific strains of *S. cerevisiae* that reside in the human gut may play an important biological role and have a major impact on human health. Validating the role of these overexpressed metabolites is beyond the scope of the present work. Notwithstanding this limitation, by combining the results of the present study with data from the literature, we hypothesize that one or more of such overexpressed molecules may play a role in the impairment effects observed on CFS-treated *C. albicans*. Further studies, including mechanistic validation of such metabolites, are warranted to test the effects of these purified metabolites on *C. albicans*. To increase the identification confidence of inosine (one of the overexpressed metabolites) from probable to possibly confirmed structure, an inosine reference standard solution was used to confirm the [M + H]+ molecular ion mass-to-charge ratio, along with its fragmentation spectrum and retention time, as detailed in previous work [55].

Notwithstanding these preliminary results obtained by in vitro studies, we hypothesize that the anti-*Candida* activity of CFSs from *S. cerevisiae* has the potential for a possible future inclusion in a postbiotic formulation. Further studies aimed at the precise characterization of the *S. cerevisiae* strains used for the production of CFSs are required, since preparations derived from undefined microorganisms cannot match the definition of postbiotics [32]. In addition, since the results presented here match the data from the literature that demonstrate anti-*Candida* effects of CFSs obtained by a pool of probiotics (including *S. boulardii*) [59], further studies are warranted to better characterize the anti-*Candida* activity of *S. cerevisiae* CFSs, as well as the overexpressed metabolites highlighted by the untargeted metabolomic analysis.

## Data Availability

The original contributions presented in the study are included in the article. Further inquiries can be directed to the corresponding author.

## Supplementary Materials

The following supporting information can be downloaded at: https://www.mdpi.com/article/10.3390/jof12020081/s1, Table S1: Compounds overexpressed in CFS 66, CFS 83, and CFS 84 with respect to CFS Ref, identified in positive ionization mode according to Compound Discoverer (CD) 3.3.2.31 analysis; Table S2: Compounds overexpressed in CFS 66, CFS 83, and CFS 84 with respect to CFS Ref, identified in negative ionization mode according to Compound Discoverer (CD) 3.3.2.31 analysis.
